# Antiviral effect of cationic compounds on bacteriophages

**DOI:** 10.3389/fmicb.2013.00046

**Published:** 2013-03-12

**Authors:** Mai H. Ly-Chatain, Saliha Moussaoui, Annabelle Vera, Véronique Rigobello, Yann Demarigny

**Affiliations:** Unité Bioengénierie et Dynamiques Microbiennes aux Interfaces Alimentaires, ISARA-LYONLyon, France

**Keywords:** bacteriophage, *Lactococcus*, MS2, chitosan, CTAB, nisin, lysozyme, virucidal activity

## Abstract

The antiviral activity of several cationic compounds – cetyltrimethylammonium bromide (CTAB), chitosan, nisin, and lysozyme – was investigated on the bacteriophage c2 (DNA head and non-contractile tail) infecting *Lactococcus* strains and the bacteriophage MS2 (F-specific RNA) infecting *E. coli*. Firstly, these activities were evaluated in a phosphate buffer pH 7 – 10 mM. The CTAB had a virucidal effect on the *Lactococcus* bacteriophages, but not on the MS2. After 1 min of contact with 0.125 mM CTAB, the c2 population was reduced from 6 to 1.5 log(pfu)/mL and completely deactivated at 1 mM. On the contrary, chitosan inhibited the MS2 more than it did the bacteriophages c2. No antiviral effect was observed for the nisin or the lysozyme on bacteriophages after 1 min of treatment. A 1 and 2.5 log reduction was respectively observed for nisin and lysozyme when the treatment time increased (5 or 10 min). These results showed that the antiviral effect depended both on the virus and structure of the antimicrobial compounds. The antiviral activity of these compounds was also evaluated in different physico-chemical conditions and in complex matrices. The antiviral activity of CTAB was impaired in acid pH and with an increase of the ionic strength. These results might be explained by the electrostatic interactions between cationic compounds and negatively charged particles such as bacteriophages or other compounds in a matrix. Milk proved to be protective suggesting the components of food could interfere with antimicrobial compounds.

## INTRODUCTION

In the dairy industry, *Lactococcus lactis* is the most common genus used for fermentations of many kinds of cheese. These bacterial species are sometimes infected by bacteriophages, which leads to the cessation of fermentations and the disappearance of several microbial populations in the ripened cheese. Bacteriophages found in raw milks originate from air-borne contaminations or machines and they can rapidly contaminate the milk or the fermentation process (Rousseau and Moineau, 2009). Studies on bacteriophage biodiversity in the dairy industry identified more than ten different lactococcal bacteriophages. Among them, three main groups: c2, P335, and 936 were most frequently detected (Deveau et al., 2006), the c2 group being the most common in cow’s milk. To avoid bacteriophage contaminations in the dairy environment, their inactivation through high pressure treatments, heat treatments or biocides have been studied (Moroni et al., 2002; Quiberoni et al., 2003; Atamer and Hinrichs, 2010). Although the physical treatment parameters have been extensively studied and defined, scientific literature contains few studies on the use of antiviral compounds, and this despite the fact that several bacteriophages are known to be resistant to pasteurization and some disinfectants such as ethanol or isopropanol. In order to find an effective treatment to reduce or eliminate bacteriophage contamination in the environment, we studied the antiviral effect of several positively charged compounds such as chitosan, cetyltrimethylammonium bromide (CTAB), nisin, and lysozyme.

Among antimicrobial substances, chitosan comes from the partial or the complete deacetylation of chitin, a component of the exoskeleton of *crustaceans,* including crabs or shrimps, many insects – spiders – and moulds. Chitosan exhibits strong antimicrobial effects against various pathogenic and spoilage organisms (Kong et al., 2010). Moreover, chitosan is biodegradable, harmless, and antioxidative. For these reasons, chitosan has been studied for many applications, especially in the food industry, to improve food safety. Chitosan has been investigated in many food products, and among them, breads, noodles, pasta, rice, eggs, and dairy products. Strangely, chitosan has never been used against viruses.

Cetyltrimethylammonium bromide is a class of aliphatic quaternary ammonium compounds which have a strong antimicrobial activity. For instance, some of these compounds have been used as disinfectant agents in the medical environment and the food industry (Sands, 1986).

Nisin and lysozyme are antimicrobial proteins. Nisin is a bacteriocin produced by lactic acid bacteria. Lysozyme is found in high quantities in egg white but also in tears, saliva, or milk. These compounds are active against many food-borne pathogens. They are widely regarded as natural biopreservatives in foods to inhibit the growth of Gram-positive cells and spores (Bower et al., 1998). These compounds, just as chitosan, have never been tested on viruses.

These compounds share a common chemical characteristic: they are positively charged. Their antimicrobial activity is thus related to their ionic charge but also to hydrophobicity and the environmental conditions (Nicoletti et al., 1993; Rabea et al., 2003; Gorbenko et al., 2007; Di Pasquale et al., 2010). These compounds can bind to the bacterial cell surface or directly to viral particles. In the case of bacteria, the positively charged compounds adsorb firstly at the surface of the negatively charged bacteria. They can harm the cell membrane directly or penetrate inside the cell to precipitate proteins (Nicoletti et al., 1993; Rabea et al., 2003; Gorbenko et al., 2007; Di Pasquale et al., 2010). The most negatively charged bacteria are the most susceptible to cationic compounds (Ly et al., 2006; Potter et al., 2005). The interactions between cells and antimicrobial compounds depend on the physico-chemical conditions and the biochemical composition of the food matrix (Devlieghere et al., 2004; Ly et al., 2006). In the case of virus, the positively charged compounds can adsorb on viral capsid by also electrostatic interaction which inhibit viral adsorption on host cells (Pan et al., 2006). However, the microbial activities of cationic compounds have mainly been focused on pathogenic bacterial but they were less studied on viruses.

In this study, the antiviral activity of chitosan, CTAB, nisin, and lysozyme was investigated. In particular, we tried to establish a link between viral charge, the antiviral activity of several positively charged compounds and the interference of the food matrix.

The resistance of the c2 lactococal bacteriophage to these different antimicrobial compounds was compared to that of the bacteriophage MS2 which is used as an indicator of enteric viruses. During the course of our experiments, MS2 was considered as a control. Enteric virus contamination in milk, food, and water is considered a major cause of incidence of food-borne diseases (Baert et al., 2009; Stals et al., 2012). The inactivation of MS2 has been studied for preservation methods against food-borne viruses as well as for water treatments (Olson et al., 2004; Yavarmanesh et al., 2010).

## MATERIALS AND METHODS

### LACTOCOCCAL BACTERIOPHAGES AND BACTERIAL STRAINS

The *L. lactis* strains MG1363, originating from institut national de la recherche agronomique (INRA) Dijon, were used as host cells of phage c2. They were stored at -80°C in a glycerol solution (10%) and cultured in M17 broth (Fluka) supplemented with lactose (0.5% as final concentration) at 30°C when needed.

The bacteriophages were added in the cultures composed of host bacteria supplemented with lactose (0.5%) and CaCl_2_ (10 mM) at an early stationary phase (optical density at 600 nm, OD = 0.7). The infected cultures were then incubated at 30°C for 5 h. After replication, the phage suspensions were inoculated at 4°C for the night before being centrifuged (5000*g *for 15 min). The supernatants were decontaminated of the bacteria by filtration through sterile membrane filters (0.45 μm, Millipore). The phage concentration was quantified using the double agar layer plaque assay method (Lillehaug, 1997). The phage stocks were stored at 4°C.

### F-SPECIFIC RNA BACTERIOPHAGE MS2

The bacteriophage MS2 was obtained from the ATCC culture collection (15597-B1) and replicated according to standard procedure (ISO 10705-1, 1995) without the CHCl_3_ lysis step and using the *E. coli* Hfr K12 (ATCC) as bacterial host. After replication, the bacteriophages were titrated as follows: 1 mL of the phage suspension and 1 mL of the bacterial culture (OD = 0.5) were added together in a test tube containing 2 mL of trypton yeast glucose agar (TYGA), a semi-solid medium (8 g/L agar) previously supplemented with a solution of CaCl_2_/glucose (0.3 mg/10 mg per L). The content of this test tube was then put onto a petri dish containing the tryptone yeast glucose broth (TYGB) medium (TYGA with 15 g/L agar). After overnight incubation at 37°C, plates were examined for plaque formation and the number of bacteriophages per mL of sample was calculated.

### ELECTROPHORETIC MOBILITY MEASUREMENTS OF BACTERIOPHAGES

The electrostatic properties of bacteriophages were assessed by microelectrophoresis. The electrophoretic mobility (EM) was determined at pH 2–7. Bacteriophages were diluted in a solution of NaNO_3_ (10 mM) to obtain a final concentration of around 10^7^ PFU/mL. pHs were adjusted by addition of KOH and HCl (100 mM). An aliquot of 1 mL of the bacteriophage solution was injected into the measurement chamber of a Zetasizer Nano ZS (Malvern, Worcs).

The EM was evaluated at room temperature on a ZM 77 Zetameter model (Zetameter Inc., New York, NY, USA). The EM, expressed in 10^-8^ m^2^ V^-1^ s^-1^, was derived from the velocity of the bacteriophages in suspension under an applied electric field of 100 mV.

### DETERMINATION OF THE ANTIVIRAL EFFECTS OF CATIONIC ANTIMICROBIAL COMPOUNDS

#### Preparation of antimicrobial compound solutions:

The cationic antimicrobial compounds under investigation were purchased from Sigma-France. They were prepared as followed:

- CTAB (hexadecyltrimethylammonium bromide; Sigma H 5882) was prepared in distilled water (5% w/v). The solution was stirred for 18 h at 30°C, filtered (0.45 μm) and stored at 4°C before use.
- Chitosan (Sigma 448877) was prepared in a solution of acetic acid (1% w/v). The solution was stirred at 30°C for 18 h, filtered (0.45 μm) and stored at 4°C.
- Nisin (Sigma N5764) was dissolved in 0.02 M HCl. The concentration was expressed in International Units per millilitre (IU/mL) with 1 mg = 40 IU nisin). The solution was stirred for 24 h at 4°C before centrifugation (10 min at 4000*g*) and filtration (0.45 μm). It was then stored at 4°C.
- Lysozyme (Sigma-62971) was prepared in distilled water. The concentration was expressed in International Units per millilitre (IU/mL) with 1 mg = 96,381 IU. The solution was stirred for 18 h at 30°C and filtered (0.45 μm) and stored at 4°C.

***Test of antiviral effect*** A stock solution of phages (approximately 10^9^ PFU/mL) was diluted in the solution test (buffer, culture media, or milk) to obtained a final concentration of 10^6^ PFU/mL. Aliquots of 5–50 μL (depending on the concentration to be tested) of antimicrobial stock solution were then added in a tube containing 1 mL of the 10^6^ PFU/mL phage solution and mixed briefly. The tube was incubated at room temperature for 1, 5, or 10 min. The phage suspension was then diluted and enumerated immediately by the double layer method described above. The control test was carried out identically without the addition of antimicrobial compounds. Three trials of each assay were carried out.

## RESULTS

### ANTIVIRAL EFFECT OF CATIONIC COMPOUNDS ON BACTERIOPHAGES

**FIGURE 1:**
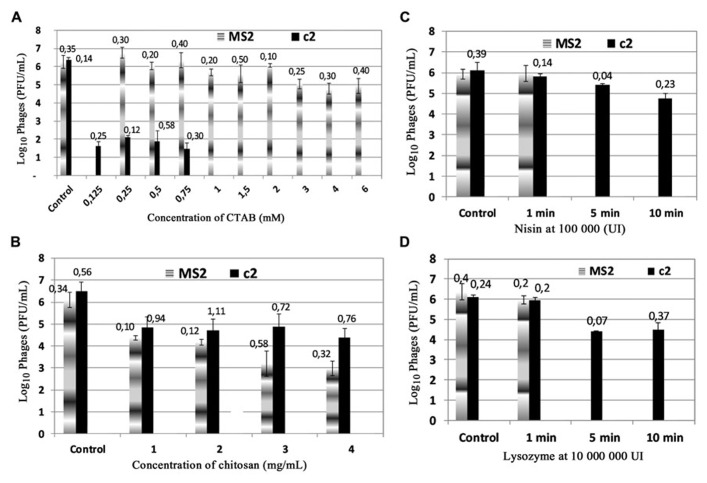
**Antiviral activities of cationic antimicrobial compounds on bacteriophages c2 and MS2: **(A)** CTAB; **(B)** chitosan; **(C)** nisin, and **(D)** – lysozyme.** The phages were incubated at room temperature for 1 min and enumerated by the double layer method. Control without antimicrobial compound. Value of ±SD is on the top of column.

The antiviral activities of the cationic antimicrobials compounds CTAB, chitosan, nisin, and lysozyme against MS2 and c2 bacteriophages were studied firstly in a phosphate buffer pH 7 – 10 mM. The **Figure 1A** shows the antiviral activity of CTAB at different concentrations varying from 0.125 to 6 mM. After 1 min of exposure to CTAB at 0.125 mM, the c2 phage population was reduced from 6 to 1.5 log(pfu)/mL and completely deactivated at 1 mM of CTAB. On the contrary, MS2 bacteriophage populations did not seem to be affected by CTAB and only lost 1.5 log(pfu)/mL after exposure to 3, 4, or 6 mM of CTAB.

The chitosan concentrations tested ranged from 1 to 4 mg/mL (**Figure 1B**). At 1 mg/mL of chitosan for 1 min of treatment a reduction of 2 log was observed for MS2 and c2. Antiviral activity on MS2 heightened as the chitosan concentration increased, but the c2 did not seem to be affected by greater concentrations of chitosan.

Nisin and lysozyme seemed to exert a weak activity on c2 and MS2 populations. Different concentrations were tested (results not shown), but they led to the same result. **Figures 1C,D** only show the antiviral activity of nisin and lysozyme at the highest concentration tested. For 1 min of contact, any reduction of phage c2 and MS2 was not affected at 100,000,000 IU of lysozyme and at 100,000 IU of nisin. When the exposure duration increased (5 or 10 min), a reduction of 2 log was observed for lysozyme and 1 log of reduction for nisin after 10 min contact.

### EFFECT OF MATRICES ON ANTIVIRAL ACTIVITY OF CTAB AND CHITOSAN

**FIGURE 2:**
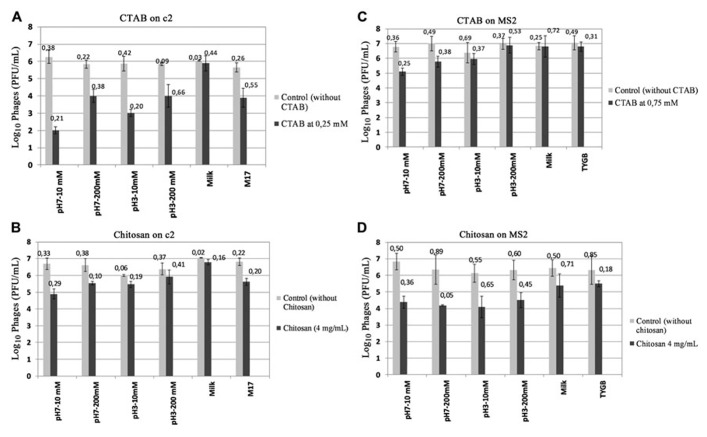
**Effect of matrices on antiviral activity: **(A)** Antiviral activity of CTAB on c2 in different matrices**. **(B)** Antiviral activity of Chitosan on c2 in different matrices. **(C)** Antiviral activity of CTAB on MS2 in different matrices. **(D)** Antiviral activity of chitosan on MS2 in different matrices. The phages were incubated at room temperature for 1 min and enumerated by the double layer method. Value of ±SD is on the top of column.

The inhibitory concentration of CTAB and chitosan on c2 and MS2 was tested in a buffer with varying pH and ionic strength, a medium culture and milk. **Figure 2** shows that in the absence of antimicrobial compounds, the infection of both bacteriophages c2 and MS2 was not affected in different matrices. When the antimicrobial compounds were tested in these matrices, the antiviral activity changed depending on physico-chemical conditions, the composition of matrices and on the kind of phage. CTAB and chitosan were less active on c2 at acid pH and at high ionic strengths (**Figures 2A,B**). For example, the antiviral activity on c2 was lowest at pH 3 – 200 mM. At pH 7, the antiviral activity at 200 mM was less than at 10 mM. Antiviral activity of CTAB and chitosan on c2 were weaker in the medium culture M17 than the buffer pH 7 – 10 mM. The c2 was completely protected in milk, any reduction of phages was observed when the antimicrobial compounds were tested in milk.

Similarly, the antiviral activity of CTAB on MS2 was reduced at pH acid and with the increase of ionic strengths. On the contrary, the antiviral activity of chitosan against MS2 was less affected by the matrix than CTAB.

### ELECTROPHORETIC MOBILITY MEASUREMENTS

**FIGURE 3:**
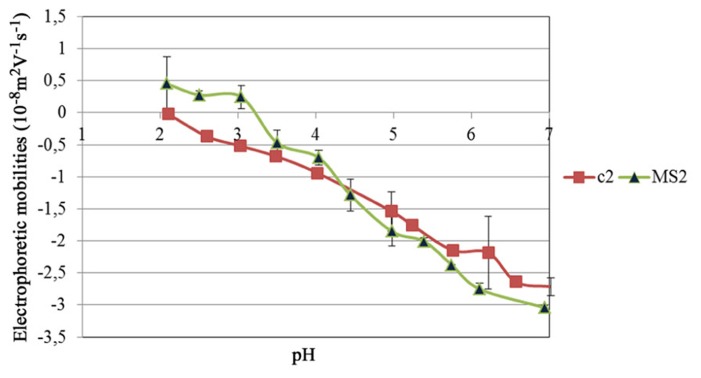
**The electrophoretic mobility of bacteriophage MS2 and c2 as function of pH in phosphate buffer**.

In order to understand the electrostatic interactions between phages and antimicrobial compounds, the EM of the two bacteriophages was evaluated in phosphate buffer 10 mM at different pH (**Figure 3**). Both bacteriophages were negative at pH values above 4 and became more positive when pH decreased below 4. The isoelectric point (IEP) of c2 was around pH 2. The IEP of MS2 was around 3.5.

## DISCUSSION

Studies on the antimicrobial activities of different compounds are carried out in the aim of finding ways to reduce microbial populations in food products, packaging, and equipment. However, information on viricidal activity is much less prevalent than that concerning bactericidal, fungicidal, and sporicidal activities. This is particularly true for chitosan, CTAB, and bacteriocins (nisin and lysozyme). Among them, chitosan is the most studied compound for its antimicrobial activity against Gram-negative and Gram-positive bacteria. The minimum inhibitory concentration (MIC) and minimum bactericidal concentration (MBC) of chitosan on bacteria vary respectively from 8 to 16 μg/mL and from 32 to 64 μg/mL (Kong et al., 2010). In our study, the MIC concentrations obtained on bacteriophages c2 and MS2 proved to be inferior (1 mg/mL).

Cetyltrimethylammonium bromide is a biocide belonging to the quaternary ammonium category. This class of molecules has been used as a disinfectant and an antiseptic for food and body surfaces. As with chitosan, available studies only deal with the bactericidal effect of CTAB. The MIC for a 1 min treatment was evaluated at 10 μM for *Salmonella typhimurium* and 50 μM for the yeast *Candida albicans *(Lindstedt et al., 1990). Bacteria were completely deactivated after a 10 min period of exposure; 30 min for the fungi. In our study, the MIC (125 μM) and the MBC (1 mM) on c2 were superior to these concentrations confirming the high resistance of viruses compared to other microorganisms (Hijnen et al., 2006). Sands (1986) have found that the rate of deactivation of two mammalian viruses (HSV-2 and SV40) was superior to 95% after an exposure to 30 μM CTAB for 15 min.

Nisin is regarded as a natural antimicrobial agent, since it is a polypeptide produced by some strains of lactic acid bacteria (*L. lactis*). This bacteriocin exerts an antimicrobial activity against Gram-positive bacteria, particularly against spores (da Silva Malheiros et al., 2010; Tong et al., 2010; Arqués et al., 2011). The antiviral activity of nisin has never been studied. However, the results of this study have shown that nisin was able to exert a slight effect on bacteriophages. The bacteriophage MIC of nisin was very high (100,000 UI) for a 10 min long treatment compared with the MIC obtained on bacteria (2,400 UI). The absence of effect of nisin on bacteriophages shows that it is possible to combine the phages and nisin to fight the pathogens (Martínez et al., 2008).

Lysozyme has been commercialized for applications such as a natural preservative to control bacteria in meat products such as sausages, salami, pork, and beef, to prevent the growth of many kinds of pathogenic bacterial (Hughey and Johnson, 1987; Deckers et al., 2008). It can also be used for other pharmaceutical applications (Memarpoor-Yazdi et al., 2012). As observed for nisin, the MIC of lysozyme against bacteriophages was also very high (1,000,000 UI) compared with the several bacterial strain (500,000 UI for 30 min exposed). In the future, it will be interesting to test the antiviral activity of nisin and lysozyme on bacteriophages by extending the time of treatment.

The antimicrobial activity of chitosan and CTAB in relation to environmental factors has been reported in the literature. Chitosan exhibited a strong inhibitory effect at low pH whereas the antimicrobial activity decreased with the increase of pH (Kong et al., 2010). On the contrary, the virucidal activity of CTAB was obtained at basic pH (Sands, 1986). Antiviral activity of CTAB at pH 3 was less than pH 7. This result could be explained by electrostatic interactions between the phages and the CTAB molecules. At pH acid, phages have a neutral or positive charge leading to a reduction the absorption of cationic compounds on phage. Vieira and Carmona-Ribeiro have also showed that the antifungal activity of cationic surfactant such as CTAB or DODAB (dioctadecyldimethylammonium bromide) release rather the cell surface charge (Débora et al., 2006). A lower cell viability have been observed when the cell surface charge change from negative to positive. But these results were not observed with chitosan, which suggests that the electrostatic interaction may be important for the contact between phages and other compounds, but other factors (e.g., the hydrophobicity of chitosan) could also have an influence on it.

Generally, the virucidal activity of cationic compounds is reduced with the increase of the ionic strength. This suggests that the cations in the medium may interact competitively with the negative charges that surround the bacteriophages. Similarly, the bacteria or virus could be more resistant to antimicrobial compounds in a complex medium such as culture medium or milk because the compounds in the matrix may interfere with the antimicrobial compounds (Devlieghere et al., 2004).

Contrary to c2, the antiviral activity of chitosan against MS2 was less affected by physico-chemical conditions or a complex matrix. The difference in antiviral activity on the two phages c2 and MS2 could be related to their capsid structure and to the mode of action of the antimicrobial compounds. c2 is a DNA phage with a contractile tail whereas MS2 is a small isometric phage. Until now, the resistance mechanisms of viruses and bacteriophages towards antimicrobial compounds have rarely been documented. In general, two antiviral mechanisms have been identified. The first one have been that antimicrobial compounds may interact with the receptor on cell surface which are the binding sites for many viruses. Other mechanism have been the binding directly of antimicrobial compounds on viral particles which inhibit viral adsorption (Pan et al., 2006). In the last case, the charge of antimicrobial compounds and viral particles play an indispensable role. If the antimicrobial compounds positively charged such as CTAB and the bacteriophages negatively charge at neutral pH, the antimicrobial compounds may adsorption on viruses or bacteriophages by electrostatic interactions which alter viral capsid structure and viral nucleic acids. However, the alteration of viral markers, such as the antigenic structure and DNA polymerase, as well as the structural damages caused to the capsid does not always reflect a loss of viral infectivity (Maillard, 2001). Several authors suggest that the viral resistance to antimicrobial compounds may be related to viral aggregation. The MS2 aggregation occurred in acid pH and in high ionic strengths (Dika et al., 2011). This could explain why MS2 was not affected by environmental factors and a complex matrix.

This study is exploratory. It has to be deepened by the investigation on other bacteriophages with various treatment times (10, 15, 30, or 60 min). The results obtained with chitosan and CTAB against c2 and MS2 already contribute to our understanding of phage resistance towards antimicrobial compounds and could help us to find new effective solutions for cleaning food products and disinfecting the equipments used. These results suggest that using quaternary ammonium detergents to disinfect equipment could play an important role in fighting against unwanted lactococcal bacteriophages.

## Conflict of Interest Statement

The authors declare that the research was conducted in the absence of any commercial or financial relationships that could be construed as a potential conflict of interest.
